# How to Succeed

**Published:** 1898-02

**Authors:** 


### How to Succeed.

The fellow that struggles most at the beginning of the race will have less at the end. Never fear disadvantages; good discipline and persistency will overcome all obstacles. Pay little attention to the roughness of the way; let this discourage the other fellow. Be thankful that final success requires a long pull, a strong pull, and a struggle all the way, because this will give you less and less competition as you successfully pass incompetents and weaklings. If you mind your own business and forge ahead, your disadvantages will decrease and your advantages increase, and your attention will be less attracted by side shows and petty cures. Your field will be clear, your position secure, and your success assured.— *Wilhelm Whinter.*

